# Prevalence of primary cardiac tumor malignancies in retrospective studies over six decades: a systematic review and meta-analysis

**DOI:** 10.18632/oncotarget.17378

**Published:** 2017-04-24

**Authors:** Shuai He, Yide Cao, Wei Qin, Wen Chen, Li Yin, Hao Chai, Zhonghao Tao, Shaowen Tang, Zhibing Qiu, Xin Chen

**Affiliations:** ^1^ Department of Thoracic and Cardiovascular Surgery, Nanjing First Hospital, Nanjing Medical University, Nanjing, Jiangsu, China; ^2^ Department of Epidemiology, School of Public Health, Nanjing Medical University, Nanjing, Jiangsu, China

**Keywords:** prevalence, malignancy, primary cardiac tumors, retrospective studies, meta-analysis

## Abstract

The incidence of patients diagnosed with primary malignant cardiac tumors (PMCTs) has increased greatly in the past few decades. Whether this rising prevalence is due to overdiagnosis or an increased malignancy rate of primary cardiac tumors (PCTs) remains unclear. Therefore, we performed a systematic review and meta-analysis of published retrospective studies to determine whether the malignancy rate has been increasing over time. Published studies containing relevant data between 1956 and 2014 were evaluated. Two authors searched for all retrospective studies that included patients diagnosed with PCT and PMCT. Two other investigators independently extracted the data, and discrepancies were resolved by consensus. A random-effects meta-analysis model and cumulative meta-analysis model were used to evaluate the pooled prevalence and trend of dynamic change in PCT malignancies. The effects of time, study period and sample size were studied using a logit-linear regression model with robust error variance and a time variable. Thirty-eight studies involving 5,586 patients were analyzed. The pooled prevalence of PMCT among the patients diagnosed with PCT was 9.9% (95% CI, 8.4% to 11.4%) (I^2^=70%; P< 0.001), and this prevalence has been stable since around 2003. In the regression model, the malignancy odds ratio remained stable from 1975 onward, and no time effect was observed. Our study confirms that PMCT is uncommon, and the prevalence of PCT malignancies remained stable in the past few decades. The clinically observed increase in incidence is unlikely to reflect a true population-level increase in tumorigenesis. This result strongly suggests that the observed increase in incidence of PMCT most likely reflects increased diagnostic detection over time.

## INTRODUCTION

Primary malignancies of the heart are extremely rare, and poor prognosis continues to challenge the diagnostic ability and surgical skill of clinicians [1–4]. According to the WHO Histological Classification of Tumors of the Heart and Pericardium, the majority of the malignant primary tumors of the heart can be categorized as various types of sarcomas, with primary cardiac lymphoma and epithelioid hemangioendothelioma accounting for a small fraction of cases [5]. The core knowledge of PMCTs is based mostly on case reports and autopsy studies due to their limited incidence and the difficulty of early diagnosis [6]. Because of the relatively small numbers and the significant referral bias of these studies, in October 2015, Oliveira and Al-Kindi [7] queried the largest cancer registry (Surveillance, Epidemiology and End Results, SEER) in the United States for all PMCTs diagnosed (>500 Patients) from 1973 to 2011 and found an increased incidence and survival of patients diagnosed with PMCT over the past 5 decades. A recent study in Italy (1998–2011) estimated the incidence of PMCTs at ≈130 per 100 million persons [8]. Whether this prevalence is a result of overdiagnosis or an increased malignancy rate of primary cardiac tumors (PCTs) is unclear. Pooling these data over similar time periods would allow for a determination of whether the malignancy rate has changed over time.

With advances in electronic imaging technology, the integrated use of cardiac imaging tools such as transthoracic or trans-esophageal echocardiography, magnetic resonance imaging (MRI) and multi-detector computerized tomography (MDCT) has become important during the diagnostic procedure [5]. These methods allow the rapid acquisition of real-time heart images with high spatial and temporal resolution and excellent characterization of the tumor tissue [9–11]. However, clinical manifestations of primary malignancies of the heart are so variable that their discovery may still be incidental during surgery or autopsy [12]. Although cardiac imaging tools are widely used and very important for clinical decision-making and surgical programs, as with other malignancies, histopathological examination is still indispensable and irreplaceable. To our knowledge, primary malignant cardiac tumors are asymptomatic until they reach significant dimensions [13], and the clinical symptoms (dyspnea, syncope, pain, arrhythmias, constitutional symptoms, and heart failure) are usually dependent on tumor size, invasiveness, friability, rate of growth, and especially, its location in the heart [14, 15].

We sought to better understand PMCTs, using retrospective studies from surgical centers around the world to investigate the prevalence of primary cardiac tumor malignancies over the past few decades.

## RESULTS

### Study

**Figure 1:**
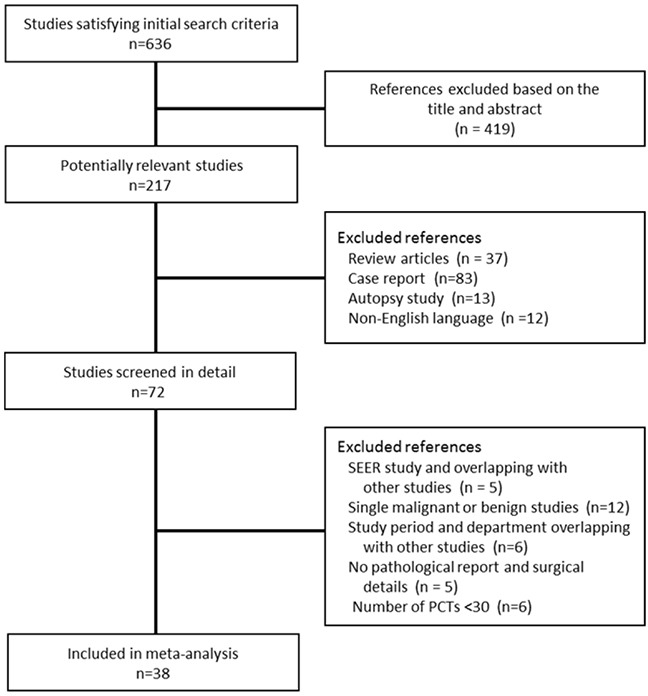
Flow diagram of study selection

We identified 636 unique publications that met the initial search criteria. The titles and abstracts were screened for inclusion. The full texts of 72 articles were retrieved, and 38 studies involving a total of 5,586 patients from 1956 to 2014 were selected and included in the final analysis (Figure 1). Characteristics of the excluded studies are listed in Table 1. Among the 38 studies, there was one registry analysis (Surveillance Epidemiology and End Results database) [7], two multicenter analyses [2, 16] and 35 single surgical center series. Twenty-seven studies performed survival analyses, and we collected the clinical outcome after surgical resection for PMCTs, which remains universally poor. The mean age of patients diagnosed with PMCTs ranged from 35.8 to 68 years of age, and 52.1% of patients were female, according to the available data. For purposes of comparing studies from different periods, we used the median year of each of the following periods when the studies were performed: before 1975 in three data sets [17–19]; between 1975 and 1980 in five data sets [2, 20–23]; between 1981 and 1985 in four data sets [4, 24–26]; between 1986 and 1990 in five data sets [5, 27–30]; between 1991 and 1995 in four data sets [31–34]; between 1996 and 2000 in eight data sets [35–42]; between 2001 and 2005 in six data sets [43–48]; and after 2005 in 3 data sets [16, 49, 50] (Table 1).

**Table 1:** Characteristics of eligible studies

Author and year	Country	Study period / median year of study period	Patients diagnosed with PMCT			No. of patients diagnosed with PCT	Malignancy rate of PCT (%)	Surgical outcome
			Mean age (years)	Female(%)	Number			
Toshiaki, 2016	Japan	2010-2013/2012	68	57	113	1317	8.6	NA
Andreas, 2015	Austria	1999-2014/2006	48.8	45.5	11	113	9.7	Late mortality (>30 d):63.3%
Barreiro, 2013	Spain	1979-2012/1995	50	45.5	11	73	15.1	1-year mortality 100%
Miralles, 1991	France	1972-1989/1981	43.5	87.5	7	73	9.6	1-year mortality 57.1%
Yu, 2007	China	1996-2005/2000	NA	NA	22	234	9.4	NA
Qingyi, 2002	China	1990-2000/1995	NA	NA	31	149	20.8	NA
Yin, 2016	China	2008-2013/2010	51	61.5	21	125	16.8	1-year mortality 70%
Dell’amore, 2013	Italy	1990-2010/2000	41	25	4	91	4.4	1-year mortality 100%
Barnes, 2014	Australia	1990-2012/2001	52	NA	6	30	20	NA
Andrew, 2008	United States	1957-2006/1982	51.42	53	19	323	6	1-year mortality 100%
Thomas, 2011	Germany	2000-2010/2005	NA	NA	3	62	4.8	1-year mortality 66.7%
Kamiya, 2001	Japan	1973-2000/1987	55.5	25	4	34	11.8	1-year mortality 75%
Blondeau, 1990	France	1961-1988/1975	46	NA	53	533	9.9	Mean survival 1.5 years
Molina,1990	United States	1959-1989/1974	NA	47.6	21	124	16.9	Mean survival 5 months
Murphy,1990	United States	1964-1989/1977	38	41.7	12	114	10.5	NA
Basso,1997	Italy	1970-1995/1983	NA	NA	9	114	7.9	NA
Centofanti, 1999	Italy	1980-1997/1989	53	80	5	91	5.5	3-year mortality 100%
Perchinsky,1997	Canada	1956-1996/1976	NA	NA	14	71	19.7	NA
Tschirkov, 1990	Bulgaria	1970-1988/1979	NA	NA	1	63	1.6	NA
Moosdoef, 1990	Germany	1971-1990/1981	NA	NA	9	51	17.6	3-year mortality 50%
Dein, 1987	United States	1961–1986/1974	NA	50	8	42	19	30-days mortality 37.5%
Grande, 1993	Italy	1980–1992/1986	35.8	0	5	31	16.1	1-year mortality 75%
Saraiva, 2016	Portugal	1994-2014/2004	55.4	66.7	12	123	9.8	1-year mortality 41.7%
Ricardo, 2014	Brazil	1986-2011/1999	44.3	50	12	185	6.5	1-year mortality < 50%
Anna, 2011	Poland	1986-2009/1998	NA	NA	5	119	4.2	1-year mortality < 20%
Massimo, 2012	Italy	1990-2005/1997	NA	NA	6	89	6.7	1-year mortality < 50%
Faisal, 2003	United States	1975-2002/1989	NA	NA	17	85	20	1-year mortality 53%
Bossert, 2005	Germany	1994-2003/1998	62.7	50	4	77	5.2	1-year mortality 50%
Patel, 2009	UK	1990-2008/1999	NA	NA	27	94	28.7	NA
Agarwal, 2003	India	1989-2001/1995	NA	NA	2	34	5.9	NA
Hoffmeier, 2005	Germany	1989-2004/1997	NA	NA	10	94	10.6	Mean survival 5.5 years
Dapper, 1988	Germany	1971-1987/1979	NA	NA	9	48	18.7	2-year mortality 88.9%
Thomas, 2007	France	1986-2005/1995	38.4	50	8	53	15.1	1-year mortality 53%
Thiene, 2013	Italy	1970–2010/1990	50	42.3	26	239	10.5	1-year mortality 88.5%
Agaimy, 2012	Germany	1999-2011/2005	45.6	60	5	74	6.7	1-year mortality 40%
Carrel, 2011	Switzerland	1996–2010/2003	NA	NA	11	155	7.1	1-year mortality 36.4%
Kumar, 2011	India	1995–2010/2002	NA	28.6	14	184	7.6	NA
Tazelaar,1992	United States	1957-1991/1974	NA	63.2	8	106	7.5	NA

PCT: primary cardiac tumor; PMCT: primary malignant cardiac tumor.

According to our risk of bias table, the most common deficiencies were the following: the relevant variables in the study populations were not closely representative of the national population; different modes of tumor examination were used in 9 studies; and nonsystematic cancer detection methods were used in 4 studies (Table 2).

**Table 2:** Quality assessment of the included studies

Author and year	External Validity				Internal Validity				
	Patients Diagnosed with PCT Were a Close Representation of the National PCT Patients	Did Not Deliberately Restrict the Included Patients in Any Way	Unavailable Data <20%	No Data Duplication	Data Collected Directly From theHospital Medical Records and Surgery Database	An AcceptableCase Definition	Cancer Detection Method Was Reliable and Valid (Histopathology)	Same Mode ofTumor Examination forAll Patients in the Study	Numerator and DenominatorMatch the Reported Results
Toshiaki, 2016	Y	Y	N	Y	Y	Y	Y	N	Y
Andreas, 2015	N	Y	Y	Y	Y	Y	Y	Y	Y
Barreiro, 2013	N	Y	Y	Y	Y	Y	Y	Y	Y
Miralles, 1991	N	Y	Y	Y	Y	Y	N	N	Y
Yu, 2007	N	Y	Y	Y	Y	Y	Y	Y	Y
Qingyi, 2002	N	Y	Y	Y	Y	Y	Y	N	Y
Yin, 2016	N	Y	Y	Y	Y	Y	Y	N	Y
Dell’amore, 2013	N	Y	Y	Y	Y	Y	Y	Y	Y
Barnes, 2014	N	Y	Y	Y	Y	Y	Y	Y	Y
Andrew, 2008	N	Y	Y	Y	Y	Y	Y	N	Y
Thomas, 2011	N	Y	N	Y	Y	Y	Y	Y	Y
Kamiya, 2001	N	Y	Y	Y	Y	Y	Y	Y	Y
Blondeau, 1990	Y	Y	Y	Y	Y	Y	N	Y	Y
Molina, 1990	N	Y	Y	Y	Y	Y	Y	N	Y
Murphy, 1990	N	Y	Y	Y	Y	Y	N	N	Y
Basso, 1997	N	Y	Y	Y	Y	Y	Y	Y	Y
Centofanti, 1999	N	Y	Y	Y	Y	Y	Y	Y	Y
Perchinsky,1997	N	Y	Y	Y	Y	Y	Y	Y	Y
Tschirkov, 1990	N	Y	Y	Y	Y	Y	Y	Y	Y
Moosdoef, 1990	N	Y	Y	Y	Y	Y	Y	Y	Y
Grande, 1993	N	Y	Y	Y	Y	Y	Y	Y	Y
Dein, 1987	N	Y	Y	Y	Y	Y	Y	Y	Y
Saraiva, 2016	N	Y	Y	Y	Y	Y	Y	Y	Y
Ricardo, 2014	N	Y	Y	Y	Y	Y	Y	Y	Y
Anna, 2011	N	Y	Y	Y	Y	Y	Y	Y	Y
Massimo, 2012	N	Y	Y	Y	Y	Y	Y	Y	Y
Faisal, 2003	N	Y	Y	Y	Y	Y	Y	Y	Y
Bossert, 2005	N	Y	Y	Y	Y	Y	Y	Y	Y
Patel, 2009	N	Y	Y	Y	Y	Y	Y	Y	Y
Agarwal, 2003	N	Y	Y	Y	Y	Y	Y	Y	Y
Hoffmeier, 2005	N	Y	Y	Y	Y	Y	Y	Y	Y
Dapper, 1988	N	Y	Y	Y	Y	Y	N	N	Y
Thomas, 2007	N	Y	Y	Y	Y	Y	Y	Y	Y
Thiene, 2013	N	Y	Y	Y	Y	Y	Y	Y	Y
Agaimy, 2012	N	Y	Y	Y	Y	Y	Y	Y	Y
Carrel, 2011	N	Y	Y	Y	Y	Y	Y	Y	Y
Kumar, 2011	N	Y	Y	Y	Y	Y	Y	Y	Y
Tazelaar 1992	N	Y	Y	Y	Y	Y	Y	N	Y

### Prevalence of PMCT

**Figure 2:**
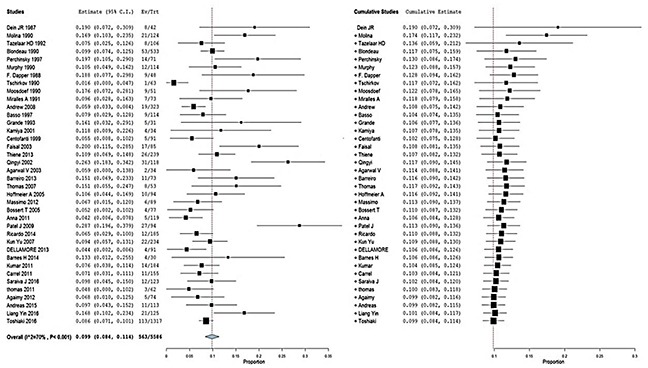
Forest plot depicting the prevalence of primary cardiac tumor malignancies **(A)** Random-effects meta-analysis model and **(B)** cumulative meta-analysis model.

**Figure 3:**
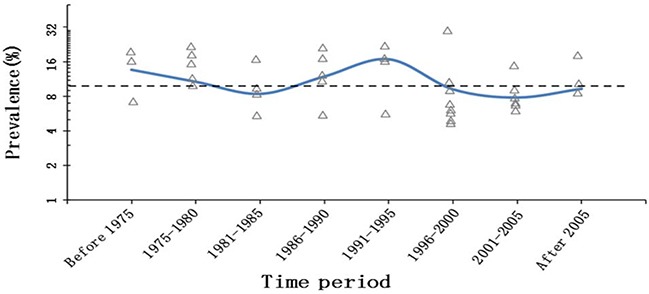
Modeled prevalence of primary cardiac tumor malignancies over time (pooled prevalence of each time period as knots) The dashed lines represent the overall prevalence of primary cardiac tumor malignancies. The y-axis is logit, and the labeled prevalence (percentage) increases are much larger in the upper part of the scale.

The pooled prevalence of malignancies among the patients diagnosed with PCT was 9.9% (95% CI,8.4% to 11.4%) (I^2^=70%; P< 0.001); similar results were determined from the cumulative meta-analysis (Figure 2). In addition, this prevalence had been basically stable since around 2003, according to the cumulative meta-analysis of all included studies (Figure 2). With the second period (1975 to 1980) as the reference, no differences in the malignancy odds were observed over the subsequent time periods. There were also no differences in the malignancy odds in relation to the study period (≤20years) and number of PCTs (≤100) (Table 3). With modeled prevalence of primary cardiac tumor malignancies, no time effect was observed from 1975 onward (Figure 3).

**Table 3:** Regression model investigating the predictors of logit prevalence of PCT malignancies

	Independent Variable	MOR	95% CI	P	I^2^
Study Period					70.40%
	Before 1975	1.03	0.94 to 1.13	0.508	
	1975-1980	1			
	1981-1985	0.99	0.91 to 1.07	0.766	
	1986-1990	1.02	0.94 to 1.10	0.706	
	1991-1995	1.05	0.97 to 1.15	0.226	
	1996-2000	0.98	0.92 to 1.05	0.572	
	2001-2005	0.97	0.91 to 1.05	0.474	
	After 2005	1.01	0.93 to 1.10	0.829	
Study Period					70.96%
	≤20 years	1			
	>20 years	0.99	0.96 to 1.04	0.993	
Number of PCTs					70.70%
	≤100	1			
	>100	0.99	0.95 to 1.03	0.637	

MOR: malignancy odds ratio; PCTs: primary cardiac tumors (relative malignancy odds of PCTs per unit increase in each predictor).

## DISCUSSION

In this study, we report the prevalence and characteristics of PMCTs using data amassed over 6 decades from large, specialized, single-center studies. We confirm the rarity and lethality of PMCTs and offer insight into their epidemiology. The data from 38 retrospective studies over similar time periods were pooled, and our results suggest that the prevalence of primary cardiac tumor malignancies has remained stable over time.

To our knowledge, the first human diagnosis of primary cardiac malignancy was reported in 1934; it was based on an electrocardiogram and biopsy of a peripheral embolic lesion [51]. As a result of the rarity of primary cardiac tumor occurrence, histological diversity, and the unique nature of heart anatomy, there have been few reports describing the epidemiology, presentation, and outcome after medical and surgical treatment [25]. Over the past several decades, our understanding of malignant cardiac tumors has gradually deepened owing to the available data from autopsy studies, case reports, and in recent years, from large, specialized, single-center studies [12]. Unless they obstruct intracardial flow or interfere with heart valve activity or the conduction system, cardiac neoplasms can remain clinically silent until they reach an advanced stage [52].

However, many of the tumors discovered during autopsy may have been incidentalomas rather than clinically significant tumors. For example, the SEER study showed that clinically apparent PMCTs have an estimated prevalence of 34 cases per 100 million persons, which is more than 100 times lower than the previous estimates from autopsy studies [7]. This study was based on clinical data; the prevalence of different cardiac tumors in this study was markedly different from that reported by the autopsy studies. Furthermore, the available data collected from surgery centers are more reliable than autopsy reports in estimating the prevalence of patients diagnosed with malignancies of primary cardiac tumors.

In 2010, Castillo and Silvay [12] reported that approximately one-quarter of all primary heart tumors are malignant according to clinical data, but available data from single-center studies vary, and the reported prevalence is unstable, which was confirmed in our report [12]. Our study determined that the prevalence of malignant PCTs has remained stable in the past few decades, but the incidence of patients diagnosed with primary malignant cardiac tumors (PMCTs) has been reported to be increased by a lot in the past few decades. The observed increase in incidence may be confined to a specific country or region, which is unlikely to accurately reflect the incidence on a population level. This also suggests that the recently observed increase in incidence of PMCT most likely reflects increased diagnostic detection over time. However, the increasing number of patients diagnosed with PCT is another possibility, which requires further study. Better survival was observed for patients diagnosed with PMCT who underwent complete surgical resection compared with patients who did not in several early studies but failed to reach statistical significance [53]. Malignant neoplasms and primary malignant cardiac tumors usually require multiple modalities of treatment. Based on the literature we have incorporated into this study, complete tumor resection, adjuvant chemo- and/or radiotherapy, palliative strategy, and even heart transplant were included as therapies [16, 37, 38, 43, 45], but the median survival of PMCT patients was less than 1 year, and this finding is consistent with previous studies that have documented survivals periods of 16.5 months and 9.6 months [26, 54]. Randomized clinical trials have not been carried out to determine the optimal therapy for these primary malignancies [12].

The limitation of our research is that due to the nature of a retrospective study, the prognosis of cardiac tumors and the longitudinal changes could not be fully assessed. Another limitation was missing data regarding the methods and the prevalence of cancer stratified by age and other factors. Our research was based on clinical data, and the patients who were not diagnosed with PMCT at the time of death are missing from our data set, which prevents the data from being reflective of the general population.

In conclusion, this study confirms the rarity and lethality of primary malignant cardiac tumors and confirms that the malignancy rate has not increased over the past several decades. It is likely that the increasing incidence of patients diagnosed with primary malignant cardiac tumor is related to improvements in cardiac imaging tools (echocardiography, magnetic resonance imaging (MRI) and multi-detector computerized tomography (MDCT)) and improvements in diagnostic technology. Additional advances should be made in diagnostic technology as well as medical records and database facilities. We look forward to seeing randomized clinical trials examining the optimal therapy for primary malignant cardiac tumors, comprehensive and in-depth understanding of these malignancies as well as the consequent benefits for the patients.

## MATERIALS AND METHODS

### Search strategy and selection criteria

PubMed, EMBASE, and Web of Science were searched for relevant studies. Searches were limited to human studies and English-language publications using the following key words: “primary cardiac tumors or tumors of the heart”, “surgical” and “experience”. To avoid missing relevant studies, references of the retrieved studies were also screened. Citation lists of the retrieved articles were manually screened to ensure sensitivity of the search strategy. Data from case reports or autopsy studies were excluded. Studies with overlapping research periods and departments were excluded. Studies in which data were limited to single malignant, benign, extracardiac and secondary cardiac tumors as well as studies involving fewer than 30 patients diagnosed with PCTs were also excluded. Two authors independently assessed studies for inclusion, and discrepancies were resolved by consensus.

### Study selection and data extraction

Data collection from the qualifying studies was independently performed by two authors (Shuai He and Li Yin). Two other investigators (Wei Qin and Zhibing Qiu) resolved any disagreements regarding the extraction of data. The following details were extracted from the eligible studies: authors; publication year; study period; study population characteristics (mean age, sex proportion, country and institution, and number of patients diagnosed with PCTs and PMCTs); and outcome (mortality or mean survival after surgical resection). Because the included studies were conducted over a range of dates, the median year of each study period was considered the time at which studies were performed.

### Quality assessment

A new risk of bias table was used to assess study quality. The tool lists common safeguards used to assess the studies; they were typically easy to apply and demonstrated high interrater agreement. The higher the number of safeguards present, the more accurate the calculation of prevalence of primary cardiac tumor malignancies. We performed nine safeguards, which included both internal and external validity items for each study. Although the included studies did not focus solely on the prevalence of PMCT, this risk of bias tool was still very applicable for quality assessment of those studies.

### Statistical analysis

A random-effects meta-analysis model and a cumulative meta-analysis model were used to evaluate the pooled prevalence and the trend of dynamic changes in PCT malignancies. We used the I^2^ to evaluate heterogeneity across studies; I^2^ > 50% correlated with high heterogeneity. A regression analysis model was used to gain additional insight into the time trend of the clinically based PMCT prevalence; the association of logit prevalence with year and other important variables that were defined as a priori was analyzed, including the period when the studies were performed (before 1975, 1975to 1980, 1981 to 1985, 1986 to 1990, 1991 to 1995, 1996-2000, 2001-2005, and after 2005), the original period of each study (≤20years and >20years), and the number of PCTs included in each study (≤100 and >100). Pooled analyses were conducted using OpenMeta-Analyst (AHRQ, grant number: R01HS018574), and the regression models were run using Stata SE version 12 (Stata Corp, College Station, TX).

## Abbreviations

PMCTs: primary malignant cardiac tumors
PCTs: primary cardiac tumors
SEER: Surveillance, Epidemiology and End Results
MRI: magnetic resonance imaging
MDCT: multi-detector computerized tomography
